# Taxonomic and Functional Diversity of Heterotrophic Protists (Cercozoa and Endomyxa) from Biological Soil Crusts

**DOI:** 10.3390/microorganisms9020205

**Published:** 2021-01-20

**Authors:** Samira Khanipour Roshan, Kenneth Dumack, Michael Bonkowski, Peter Leinweber, Ulf Karsten, Karin Glaser

**Affiliations:** 1 Institute for Biological Sciences, Applied Ecology and Phycology, University of Rostock, Albert-Einstein-Str. 3, 18059 Rostock, Germany; ulf.karsten@uni-rostock.de (U.K.); karin.glaser@uni-rostock.de (K.G.); 2 Institute of Zoology, Terrestrial Ecology, University of Cologne, Zülpicher Str. 47b, 50674 Cologne, Germany; kenneth.dumack@uni-koeln.de (K.D.); m.bonkowski@uni-koeln.de (M.B.); 3 Faculty of Agriculture and Environmental Sciences, Soil Science, University of Rostock, Justus-von-Liebig-Weg 6, 18059 Rostock, Germany; peter.leinweber@uni-rostock.de

**Keywords:** Cercozoa, eukaryvory, feeding behavior, functional traits, soil food web

## Abstract

Biological soil crusts (biocrusts) accommodate diverse communities of phototrophic and heterotrophic microorganisms. Heterotrophic protists have critical roles in the microbial food webs of soils, with Cercozoa and Endomyxa often being dominant groups. Still, the diversity, community composition, and functions of Cercozoa and Endomyxa in biocrusts have been little explored. In this study, using a high-throughput sequencing method with taxon-specific barcoded primers, we studied cercozoan and endomyxan communities in biocrusts from two unique habitats (subarctic grassland and temperate dunes). The communities differed strongly, with the grassland and dunes being dominated by Sarcomonadea (69%) and Thecofilosea (43%), respectively. Endomyxa and Phytomyxea were the minor components in dunes. Sandonidae, Allapsidae, and Rhogostomidae were the most abundant taxa in both habitats. In terms of functionality, up to 69% of the grassland community was constituted by bacterivorous Cercozoa. In contrast, cercozoan and endomyxan communities in dunes consisted of 31% bacterivores, 25% omnivores, and 20% eukaryvores. Facultative and obligate eukaryvores mostly belonged to the families Rhogostomidae, Fiscullidae, Euglyphidae, Leptophryidae, and Cercomonadidae, most of which are known to feed mainly on algae. Biocrust edaphic parameters such as pH, total organic carbon, nitrogen, and phosphorus did not have any significant influence on shaping cercozoan communities within each habitat, which confirms previous results from dunes.

## 1. Introduction

Globally distributed biocrusts entail an aggregation of soil particles and host diverse communities of terrestrial phototrophic and heterotrophic organisms [1,2]. Heterotrophic bacteria, phototrophic cyanobacteria, and green algae, along with fungi, stabilize the bare soil surface and establish a community that is known as early biocrusts [3]. In the later successional stages, lichens and mosses grow [1]. Accordingly, biocrusts can reinforce the soil surface against erosion and increase soil fertility by nutrient inputs from primary production and nitrogen fixation [4,5], which facilitate subsequent vascular plant growth [6].

Biocrust consumers such as heterotrophic protists, rotifers, tardigrades, nematodes, and micro-arthropods are vital players in the soil food web [2]. While phototrophic organisms function as primary producers, heterotrophic soil protists represent an essential link from lower to higher trophic levels and further enable nutrient recycling through the microbial loop [7,8,9].

Cercozoa and Endomyxa are considered as dominant groups of protists in soil ecosystems [10,11,12,13]. They are morphologically and ecologically highly diverse, encompassing testate and naked amoebae, flagellates, and amoeboflagellates, which can be autotrophs, heterotrophs, and parasites. Most terrestrial representatives are known to feed on bacteria, but several taxa among them feed on eukaryotes, such as fungi and algae [14,15].

Several studies were done on the diversity and community composition of Cercozoa in various soil habitats [10,14,16,17,18]. Fiore-Donno et al. [19] were the first to evaluate the diversity of Cercozoa and Endomyxa in biocrust samples from different regions with barcoded primers specific for this group. In biocrusts from the Atacama Desert (Chile), Arctic tundra (Spitsbergen), and temperate forests (Germany), they detected cercozoan sequences that comprised nearly the whole range of the phylum. However, the study design did not facilitate a statistical comparison of habitats, and cercozoan functioning was not assessed [19]. In a recent study conducted in the coastal dunes of the Baltic Sea, using a primer-independent method, Khanipour Roshan et al. [20] showed that Cercozoa were one of the dominant heterotrophic protist groups in young algal and cyanobacterial biocrusts. However, their trophic roles in biocrusts remained largely unknown.

The current study continues the previous work of Khanipour Roshan et al. [20] by focusing on the Cercozoa and Endomyxa in two unique habitats: biocrusts in dunes from Germany (with a temperate continental climate) and biocrusts in the grassland on Iceland (with subpolar oceanic climate). Despite the substantial differences between the studied habitats as a matter of geography, climate, soil parent materials, and genesis, cercozoan communities in both habitats developed under harsh environmental conditions. Icelandic soils, due to their volcanic origin, are physicochemically different from most soils in the mainland of Europe [21]. Moreover, these soils are shaped by strong environmental processes such as glacial activity, cryoturbation, continual volcanic eruptions, and subarctic climatic conditions [22]. Coastal dunes, on the other hand, are extreme ecosystems in the transition zone between terrestrial and marine environments, where interactions between geology, climate, and vegetation create highly dynamic habitats. These harsh habitats are exposed to a wide variety of environmental stressors such as strong wind and substrate mobility, scarcity of nutrients and soil water, high temperature fluctuations near the surface, intense radiation, flooding by salt water, and salt spray [23]. In habitats of these regions where vegetation is poor and sparse, the soil surface is covered by biocrusts [24,25,26]. Studying the role of protists in these biocrusts will provide new insights into the trophic structure of their soil food web and nutrient transformations. 

In this study using a high-throughput sequencing approach with specific barcoded primers [19], we assessed the cercozoan and endomyxan communities in biocrusts from two climatically distinct habitats; coastal dunes in Germany and grassland in Iceland. We hypothesized that cercozoan diversity in biocrusts in nutrient-poor habitats like dunes differs from that of more fertile, well-developed soils such as in subarctic grassland. To test this hypothesis, the influence of biocrust chemical properties (pH, total organic C, total N, and total P) on shaping cercozoan and endomyxan communities was studied. Since biocrusts are rich in various algae, we expected a higher proportion of algivores among eukaryvorous Cercozoa and Endomyxa, who may feed mainly on algae, as well as on fungi, and also other protists. In order to clarify their feeding behavior, we determined the functional traits of the cercozoan and endomyxan communities and compared their prey spectra between habitat types.

## 2. Materials and Methods

### 2.1. Study Area and Site Description

The studied areas are located in Germany (Baltic Sea coastline) and Iceland. Biocrust samples were collected in dunes along the Baltic Sea shoreline of the German Federal State of Mecklenburg-Western Pomerania. The Baltic Sea coastline is influenced by a temperate continental climate. Climate parameters for the area were obtained from two nearby meteorological stations (Warnemünde and Karlshagen) [27]. Total precipitation was 738.6 and 689 mm for Warnemünde and Karlshagen, respectively. The recorded mean temperature for Warnemünde and Karlshagen were 10 °C and 9.2 °C, respectively. The sand dune area was covered by young cyanobacterial-algal biocrusts and sparse beach grasses.

The sampling locations in Iceland were established around Litla-Skard, which is situated in the west of the island, about 100 km north of Reykjavík. Iceland is a volcanic island with a subpolar oceanic climate featuring an annual average temperature of 3.1 °C. The average annual precipitation in the form of rain and snow is 930 and 100 mm, respectively (data are from the climate station Borgarnes) [28]. The vegetation consists of shrub birches, moss heaths, marsh grass, and grassland, as well as young cyanobacterial-algal biocrusts.

### 2.2. Sampling Design

Biocrusts sampling took place in Germany (in summer, June 2017) and Iceland (in summer, July 2014). Along the Baltic Sea shoreline of the German Federal State of Mecklenburg-Western Pomerania, twenty samples of cyanobacterial-algal biocrusts were collected in coastal dunes on sun exposed slopes (five sampling locations, four replicates from each location, Supplementary Figure S1a and Table 1).

From Iceland, twenty cyanobacterial-algal biocrusts (five sampling locations, four replicates from each location) were sampled (Figure S1b and Table 1) [29]. The five sampling sites (Table 1) represented a catena of around 60 km distance from the sea to inland and were located at different altitudes from 10 to 157 m above sea level.

For biocrust sampling, a column soil sampler (collector) was pushed 1 cm deep into the respective biocrusts and carefully lifted. Samples were gently transferred from the sampler to centrifuge tubes and immediately frozen in the field.

### 2.3. DNA Extraction and Amplification, Illumina Sequencing

DNA was extracted from about 0.25 g biocrust by using the MoBio Power Soil DNA isolation kit (MoBio Laboratories, Carlsbad, CA, USA) and kept frozen until processing. 

To amplify a DNA fragment of about 350 base pairs from the variable nuclear 18S V4 region, a two-step PCR with barcoded primers specifically designed for Cercozoa was performed [30]. In the first PCR, a mixture of the forward primers S615F_Cerco and S615F_Phyt, 50% each, and the reverse primer S963R_Phyt were used. One microliter of ten times diluted DNA was used as a template for the first PCR, and 1 µL of the resulting amplicons were used as a template for the following semi-nested PCR. In the second PCR, barcoded primers (barcode combinations are listed in Table S1), the same forward primers as in the first step, and reverse 947R-Phyt were used. The following final concentrations were used for PCR: Dream Taq polymerase (Thermo Fischer Scientific, Dreeich, Germany) 0.01 units, Thermo Scientific Taq green Buffer 1×, dNTPs 0.2 nM, and primers 1 µM. The thermal program was set to 95 °C for 2 min, 24 cycles at 95 °C for 30 s, 52 °C for 30 s, 72 °C for 40 s; and a final elongation step at 72 °C for 5 min. PCRs were carried out twice to increase the chance for some DNAs to be duplicated and to reduce the artificial dominance of few amplicons. Amplified products were then pooled together [30].

As an internal standard, a mock community from known species of Cercozoa (ten cultures) was created for the bioinformatics pipeline (as described in [14,19]). After checking the amplicons by electrophoresis, a purification and normalization step was done for 25 µL of the pooled PCR products by SequalPrep Normalization Plate Kit (Invitrogen GmbH, Karlsruhe, Germany). The process was followed by pooling all the samples and the mock community to prepare a single library. The library preparation and paired-end MiSeq sequencing with the MiSeqv3 2 × 300 bp kit were carried out by the Cologne Centre for Genomics (CCG).

Paired reads were assembled using MOTHUR v.3.9 [31], allowing one difference in the primers, no difference in the barcodes, no ambiguities, no mismatches higher than two, and removing assembled sequences with an overlap < 200 bp. Reads were sorted into samples according to the barcodes (Table S1). The quality check and removal/cutting of low-quality reads were conducted with the default parameters. Using BLAST+ [32] with an e-value of 10^−50^ and keeping only the best hit, sequences were identified in the PR2 database [33], and noncercozoan sequences were removed. Chimeras were identified using UCHIME [34] as implemented in MOTHUR, with a penalty for opening gaps of −5 and a template for aligning operational taxonomic units (OTUs, V4 region of 78 cercozoan taxa, see [19]). Sequences were clustered using VSEARCH v.1 [35], with abundance-based greedy clustering (agc) and a similarity threshold of 97%, as indicated by analyzing the mock community. The expected ten OTUs could be retrieved at 97% similarity when deleting OTUs represented by less than 4% of sequence reads, and this was set as a cutoff threshold for OTU delineation [19]. Finally, the trophic structure of Cercozoa was determined based on Dumack et al. [15]. In their study based on nutrition modes, organisms were categorized as bacterivores, eukaryvores (which feed on fungi, algae, microfauna and other protists), omnivores (feeding on bacteria and eukaryotes), and parasites.

### 2.4. Determination of Biocrust Chemical Properties

In the current study, the following chemical properties were determined in the biocrust samples. The pH was measured in a 1:2.5 soil/aqueous CaCl_2_ (0.01 M) solution. Total organic carbon (TOC), total nitrogen (TN), and total phosphorus (TP) were determined from dried and milled biocrust material. TOC and TN were determined after acidification (10% HCl) to remove inorganic C, using a CHNS-Analyzer (VARIO EL III, Elementar Analysensysteme, Hanau, Germany) [36,37]. TP was measured photometrically according to Berthold et al. [38] after digestion of the milled biocrust powder in acid persulphate (1.5 mL) (containing 5 g K_2_S_2_O_8_ (0.2 mM) and 5 mL 9 N H_2_SO_4_ (50%) in 100 mL ultrapure water) in an oven (90 °C) for 24 h. After neutralization with 1 N HCl, the samples were alkalized with nitrophenol (0.8 g in 100 mL distilled water) (two to three drops), titrated with NaOH (1 M), and HCl (1 M), filled up to 100 mL with ultrapure water and filtered (25 mm, Whatman). With a spectral photometer (Hach-Lange, DR 3900, Düsseldorf, Germany), TP was measured at 885 nm [38] in comparison to reference standards. TOC, TN, and TP from the Icelandic grassland samples, analyzed with the same methodological approaches, were provided by Pushkareva et al. [29] (Supplementary Table S2).

### 2.5. Statistical Analyses

All statistical analyses were conducted using the R software, version 3.6.1 [39]. Prior to analyzing, a table of the frequency of OTUs for each sample was generated and normalized by dividing the number of each OTU by the total number of OTUs in each sample to remove a possible bias induced by differences in the sequencing efforts.

To display cercozoan and endomyxan abundance on different taxonomic levels, a Sankey diagram was produced using the website http://sankeymatic.com/build/. Percentage of shared and unique OTUs were calculated and plotted in a Venn diagram with the “gplots” package [40]. For comparing the alpha diversity, we calculated the Shannon index and Pielou’s evenness for each sample and split the data by habitat (dunes and grassland). We tested the samples for normal distribution applying the Shapiro–Wilk test, and for homogeneity of variances applying the Levene test and Shannon indices and evenness values for both habitats met the requirements for an analysis of variance (ANOVA).

The beta diversity of the communities was analyzed by the “Vegan” package in R [41]. Dissimilarities of the Cercozoa and Endomyxa community composition were visualized by ordination based on non-metric multidimensional scaling (NMDS) using the Bray–Curtis dissimilarity index [42]. The number of iterations used to reach the best result was set to a maximum of 999 [41]. Detected dissimilarities were tested for statistical significance by PERMANOVA test. The influence of biocrust chemical properties on cercozoan beta diversity was investigated by PERMANOVA tests (with adonis function in Vegan).

## 3. Results

### 3.1. Cercozoan Community Structure, Alpha, and Beta Diversity

**Figure 1:**
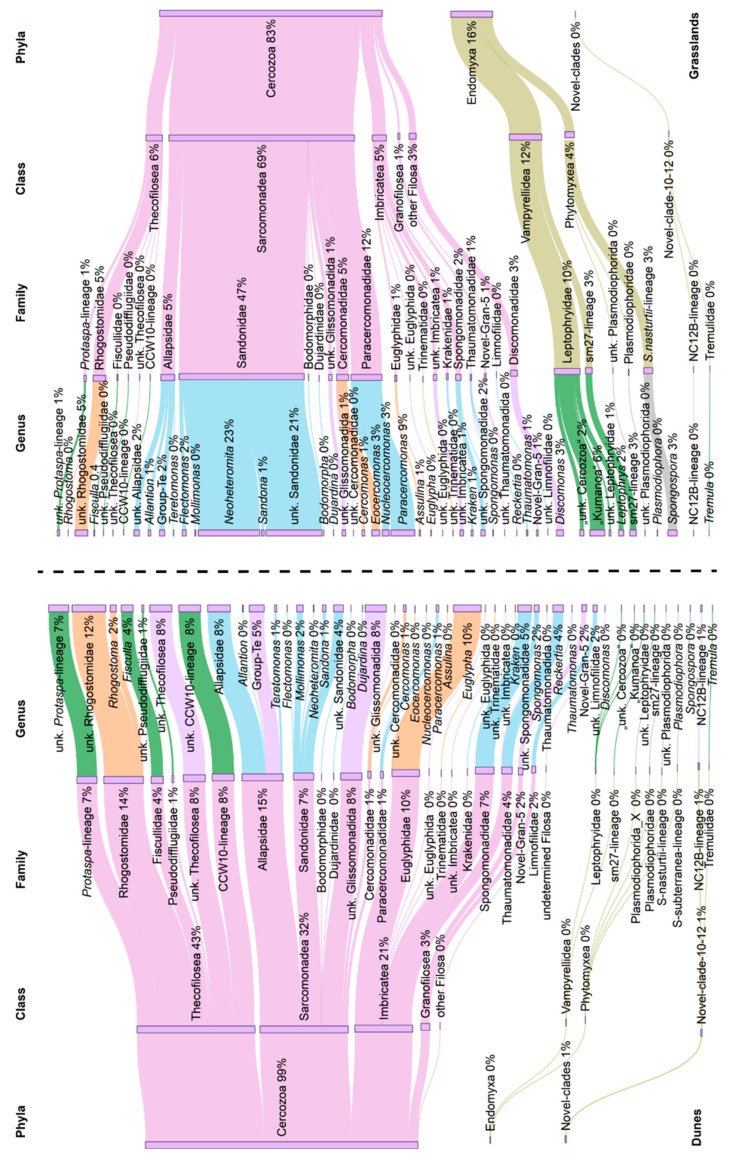
Sankey diagram based on the relative abundance of the operational taxonomic units (out) reads in biocrust samples from Germany (on the **left**) and Iceland (on the **right**). Taxonomical assignment was based on the best hit by BLAST. “Undetermined” refers to sequences that could either not be assigned to the next lower-ranking taxon or made up less than 1% of cercozoan diversity. At the genus level, the functionality (feeding behavior) of Cercozoa and Endomyxa is shown by color codes; blue color refers to bacterivores; sandy brown to omnivores; green to eukaryvores; gray to plant parasites; sequences that could not be assigned to taxa with known traits are shown in purple. Omnivorous and eukaryvorous Cercozoa include potential and known algivores.

We identified a total of 109 OTUs out of the initial 10,376,099 sequences that passed our quality filters (see Methods). At a high taxonomic level, 85% of the sequences could be assigned to Cercozoa, 13% to Endomyxa, and 2% to novel-clade-10-12. Both habitats, the subarctic grasslands and temperate coastal dunes, shared 96.3% of OTUs, with only two unique OTUs (1.8%) detected in each (Supplementary Figure S2), but the contributions of taxa in the subarctic and temperate biocrust communities differed profoundly. In biocrusts from the grassland, the cercozoan communities were dominated by the order Glissomonadida in the class Sarcomonadea (69%), followed by Endomyxa (16%). In contrast, the dominant cercozoan taxa in dunes belonged to the order Cryomonadida in the class Thecofilosea accounting for 43% of sequence reads, followed by Sarcomonadea (32%) and Imbricatea (21%). Reads assigned to Vampyrellidea and Phytomyxea (both in Endomyxa) were detected in dunes in very low abundances of between 0.1 and 0.05% of sequence reads (Figure 1). Overall, OTUs assigned to *Neoheteromita* sp. was the most abundant taxon, followed by unknown Sandonidae and Rhogostomidae (Figure 1).

At the family level, in the dune biocrusts, Rhogostomidae and Allapsidae occurred in almost similar proportions of 14% and 15%, respectively, followed by Euglyphidae (10%), the CCW10-lineage, unknown Thecofilosea, and undescribed Glissomonadida (8% each) (Figure 1). In contrast, in the grassland biocrusts, Sandonidae was the most abundant group at 47%, followed by Paracercomonadidae (12%) and Leptophryidae (10%). Some taxa (e.g., Bodomorphidae and Dujardinidae) were detected in very low abundance (less than 0.1%) in both grassland and dunes samples. 

**Figure 2:**
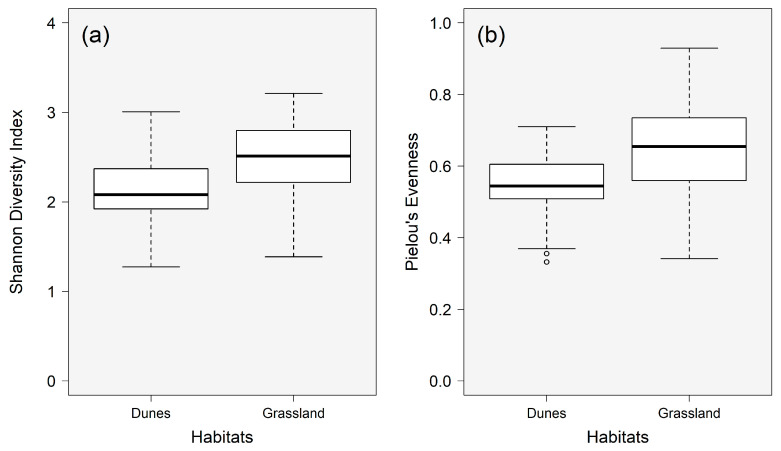
(**a**) Shannon diversity index and (**b**) Pielou’s evenness of cercozoan and endomyxan communities in biocrusts from coastal dunes (Germany) and subarctic grassland (Iceland) (significant differences between habitats, *p* = 0.03 and *p* = 0.02 for Shannon index and evenness, respectively). Individual points outside quartiles are plotted as outliers of the data.

The cercozoan and endomyxan communities in the dune and grassland biocrusts significantly differed in their alpha diversity (the Shannon diversity index (H’), H’dunes = 2.14, H’grassland = 2.43) (F = 4.93, *p* = 0.03) (Figure 2a). While OTU richness of the communities was almost similar, the evenness of the grassland communities was significantly higher than that of the dune communities (F = 5.65, *p* = 0.02) (Figure 2b). Variations in the Shannon index were also high among sampling locations both in the dune and grassland biocrusts (*p* = 0.08 and 0.05, respectively) (Supplementary Figures S3a and S4a). At the same time, variations in evenness within each habitat were non-significant (*p* > 0.05) (Supplementary Figures S3b and S4b).

**Figure 3:**
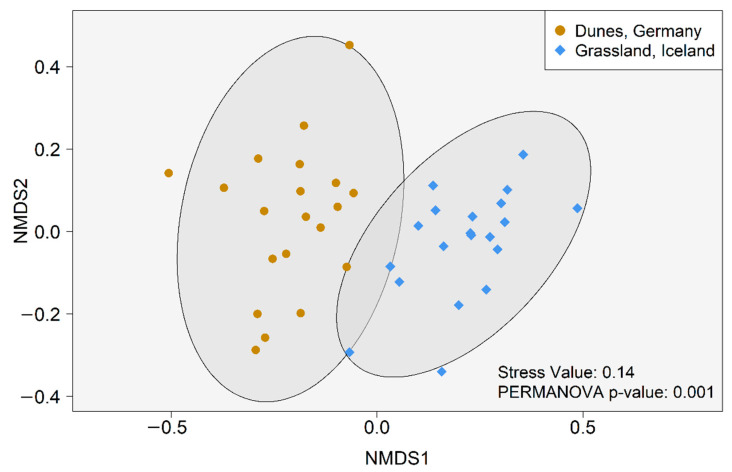
Non-metric multidimensional scaling (NMDS) based on Bray–Curtis dissimilarities among 40 biocrust samples from temperate coastal dunes (Germany) and subarctic grassland (Iceland).

The beta diversity of cercozoan and endomyxan communities differed clearly between the grassland and dune habitats (PERMANOVA, F = 6.65, R^2^ = 0.14, *p* = 0.001, Figure 3). The PERMANOVA test showed that both the organic C (F = 1.33, R^2^ = 0.08, *p* = 0.02) and the CN ratio (F = 1.55, R^2^ = 0.09, *p* = 0.007) have a significant influence on the cercozoan and endomyxan community composition between these two habitats. Noteworthy, the factors organic C and CN ratio also differed clearly between Iceland and the German dunes (Supplementary Table S2). Within each habitat, however, the PERMANOVA test showed no significant influence of biocrust chemical properties (pH, C, N, and P) on the cercozoan and endomyxan community composition among the different sampling locations (Supplementary Figure S5a,b).

### 3.2. Feeding Behavior of Cercozoa and Endomyxa

Feeding preferences of Cercozoa differed strongly between the habitats (F = 5.04, R^2^ = 0.11, *p* = 0.006). Biocrusts of the grasslands were dominated by bacterivores (70%) (e.g., Sandonidae, Paracercomonadidae), while only 31% of the cercozoans in dune biocrust were bacterivores. The communities showed a comparatively high level of omnivory (11% and 25%, in the grassland and dune biocrusts, respectively) and eukaryvory (14% and 20%, in the grassland and dune biocrusts, respectively). Omnivory was mostly related to the families Rhogostomidae, Euglyphidae, and Cercomonadidae, and eukaryvory to the families Fiscullidae, *Protaspa*-lineage and, Leptophryidae. Omnivorous and eukaryvorous Cercozoa included potential and known algivores. Plant parasites (Phytomyxea) constituted 4% of the grassland communities, while in the dune communities, this trophic group was found in very low abundance (Figure 1).

## 4. Discussion

### 4.1. Cercozoan and Endomyxan Diversity and Community Composition

The high-throughput sequencing approach with taxon-specific primers [19] allowed a thorough analysis of cercozoan and endomyxan diversity and showed that the same OTUs could be found at the Baltic Sea coast and in Iceland. This is in agreement with a recent study of Cercozoa and Endomyxa in different forest and grassland sites across Germany, which showed that, in principle, all taxa occurred everywhere [30,43]. However, the OTU richness in the coastal dune and grassland biocrusts was much lower than in the previous studies. Although the OTU richness in the studied communities was similar, the relative abundances of the same taxa in these communities were different, which explains the differences in alpha diversity, evenness, and beta diversity.

In the grassland, the orders Glissomonadida and Vampyrellidea dominated the cercozoan communities. The dominance of Glissomonadida in the grassland is well confirmed [14,17,30], but the high relative abundance of Vampyrellidea was surprising. In contrast, the communities of dunes were dominated by the orders Cryomonadida, Glissomonadida, and Euglyphida. It is possible that different microclimatic and soil conditions, ecological preferences of species, and plant communities [10,43,44] gave different taxa a competitive advantage in the grasslands and dunes. It is noteworthy that protists (e.g., cercozoan species) are able to adapt to climate conditions in their habitat and, under unfavorable conditions, can encyst and survive for a long time [44,45].

Many studies assessing the microbial composition of terrestrial habitats point to edaphic parameters such as moisture [10,14,46], clay content [14], pH, and organic nutrient availability [46] as strong environmental filters for the composition of communities. However, in agreement with Khanipour Roshan et al. [20], we found no significant influence of biocrust chemical properties on the community composition of biocrust cercozoans and endomyxans within each habitat. Total amounts of nutrients in biocrusts do not well reflect the amount of available nutrients, which are directly relevant for microorganisms [47]. Also, the harsh environmental conditions may cause high stochasticity of light exposure and soil moisture [10,14,48,49]), as reflected in the high variability of alpha diversity between sites. Nevertheless, on a geographical level, soil organic carbon, and CN ratio seem to influence the difference in cercozoan and endomyxan community composition between the grassland and dune habitats. This was in agreement with previous studies, which showed a decrease of microbial biomass with increasing CN ratios [30,50,51]. Our result may be explained by the different magnitudes of the values for soil organic carbon and CN ratios between the grassland and dune habitats (Supplementary Table S2). However, these differences, cannot be readily considered as the main driving factor of the different community composition, as they are correlated with other parameters, such as soil type and climate conditions.

### 4.2. Dunes and Grasslands Accommodate Different Bacterivores and Algivorous Cercozoa and Endomyxa

Biocrusts are a self-sustaining micro-ecosystem with a vast variety and a high abundance of algae as primary producers, e.g., in [52,53,54,55,56]. In both, dune and grassland habitats, we observed high abundances of bacterivores, which shows that bacteria are essential food sources for heterotrophic protists. Little is known whether bacterivorous terrestrial Cercozoa and Endomyxa also feed on cyanobacteria because most terrestrial cyanobacteria are filamentous and form mucilage sheds, which protect them from feeding pressure by small protists.

Our study showed that the small gliding bacterivorous Glissomonadida are highly abundant in both studied habitats in Iceland and in the Baltic Sea coastal dunes. This is in accordance with numerous other studies, likely since the Glissomonadida reproduce rapidly and, due to their small body size, generally dominate terrestrial flagellate communities. Globally, they are widespread, occurring in different habitats of temperate and tropical regions [10,16,45,57] and heathlands [17]. Recent studies showed their high abundance in biocrusts from different biogeographic regions [19] and at different soil depths [18]. Hence, it is suggested that glissomonads have a critical role in the Earth’s carbon cycling and soil food web, and probably regulate bacterial abundance on a large scale [19,45]. Remarkably, Glissomonadida in subarctic grassland biocrusts were dominated by *Neoheteromita globosa*, whereas, in dunes, they were dominated by OTUs assigned to Allapsidae and unknown taxa. This may be due to the different composition of bacterial communities in Icelandic and German biocrusts, and the possible presence of root exudates in the grassland, which favors bacteria and their predators [49,58], although the vegetation was sparse and avoided in the sampling process.

In comparison to other habitats [14,17,18,19,30], we found a high proportion of eukaryvorous Cercozoa and Endomyxa in the biocrusts from dune and grassland habitats. Various studies on different thecofilosean Cercozoa showed that most taxa appear to feed mainly or exclusively on algae, while bacterivory, and fungivory may be the exception [59,60,61,62,63,64,65,66]. Recently, Seppey et al. [65] observed a high abundance of *Rhogostoma*-assigned sequences and their strong correlation with the abundance of algae-assigned sequences in cultivated soils (e.g., meadows and croplands). According to Seppey et al. [65], species of the genus *Rhogostoma* may be the most abundant terrestrial algivorous protists. Therefore, it is not surprising that the third-most abundant cercozoan taxon in the present study was the algivorous Rhogostomidae (Thecofilosea), which was found in dune biocrusts with a proportion of 14%.

Less abundant thecofilosean taxa in our survey were undescribed species of the Protaspididae, the CCW10_lineage, Fiscullidae, and Pseudodifflugiidae. Although the prey range of these taxa has not yet been determined, from their evolutionary position, it is likely that they feed on algae as well.

In comparison with other studies [14,67], the family Leptophryidae and sm27-lineage (from the order Vampyrellidea) were more abundant in the grassland biocrusts than the dune biocrusts with 10% and 3% of the sequence reads, respectively. The vast majority of studied Vampyrellidea are obligatory algivores, although there is scattered laboratory-based evidence that some species may feed on fungi or soil mesofauna [68,69,70,71]. Many Vampyrellidea are known to be able to perforate cell walls and are thus able to prey on filamentous green algae, a food source that is inaccessible for most phagotrophic protists. For instance, the previously mentioned Rhogostomidae and other Thecofilosea ingest whole prey cells and thus feed on single-celled algae. In our study, Vampyrellidea were found in very low abundance in biocrusts from the dunes, where Rhogostomidae (and other Thecofilosea, Fiscullidae, and Pseudodifflugiidae) dominated. This may hint at different algal or cyanobacterial organization forms or shapes (filamentous vs. single-celled) in our biocrust samples, which could attract different predators or cause certain competition between Thecofilosea and Vampyrellidea. For instance, in the grassland biocrusts, diatoms, eukaryotic microalgae (such as Xanthophyceae and Klebsormidiophyceae), and filamentous cyanobacteria were observed as constituting the majority of the algal and cyanobacterial community [29]. In dunes, filamentous algae of the Klebsormidiophyceae or cyanobacteria were dominant, while single-celled algae mostly belonging to Chlorophyceae could also be observed [55,72].

Another possible explanation of competition among taxa is the ecological preferences and adaptations of Cercozoa to habitat conditions, in a way that the theca (shell) of Thecofiloseans well protects against evaporation. This has long been hypothesized and was finally supported by a strong negative correlation between soil moisture and the abundance of sequence reads assigned to shell-bearing amoebae in Fiore-Donno et al. [14]. As a consequence, in drier habitats, like pure sand in dunes with low water holding capacity in combination with high evaporation due to insolation and frequent wind, Thecofilosea may outcompete other algivorous protists that do not bear a shell, like the Vamyprellida. In contrast, Iceland biocrusts are developed mainly on Andosol, which is characterized by its origin from volcanic ashes and has a higher water holding capacity than dunes [73].

In the current study, another dominant group belonged to the omnivorous order of Euglyphida [14]. Traditionally, Euglyphida are known to also inhabit soil and freshwater, but have been mainly observed in wet mosses and bogs [74]. We found this group to be abundant in dune biocrusts, which are a rather temporarily dry habitat. This result could be explained by an increased fitness of shell-bearing taxa under drier conditions [14]. Other omnivorous families, such as the Cercomonadidae were mostly found in the grassland biocrusts. This could also be related to the prey composition because the grassland biocrusts contained diverse diatoms, filamentous algae, cyanobacteria, fungi, and bacteria [29,75].

The exceptionally low abundance of plant parasites sequences (Phytomyxea), especially in coastal dunes, can be explained by the low abundance of roots from vascular plants, whereas in the grassland with some coverage of higher plants, low abundances of OTUs assigned to *Spongospora nasturtii*, *Spongospora* sp., and *Plasmodiophora brassicae* could be identified [14].

## 5. Conclusions

In this study, we discovered diverse communities of Cercozoa and Endomyxa in biocrusts from two contrasting habitats using a high-throughput sequencing method. In both habitats, we observed similar OTU richness but different alpha and beta diversity, and evenness. The composition of cercozoan and endomyxan communities within each habitat did not change under the influence of biocrust chemical properties, as at higher trophic levels, dependency on edaphic factors decreases. This suggests that other factors are responsible for shaping the community, such as prey communities or other habitat properties (e.g., soil density/compaction and nutrient status). As the OTU richness was found to be similar, it is likely that different microclimatic conditions in dunes and grassland caused most of the detected taxa to grow in different proportions. At a geographic level, soil organic carbon and CN ratio influenced the cercozoan and endomyxan community composition between the grassland and dune habitats, which suggests these factors as possible drivers for the observed difference.

The dominance of bacterivores in grassland biocrusts suggests bacteria as the main food source, which growth is known to be stimulated by root exudates of the dominant vegetation. It seems that in dunes, a variety of food sources such as algae created an opportunity for a more pronounced community of algivores. For a better understanding of eukaryvory in biocrust food webs, the co-occurrence of algal and other eukaryotes (e.g., fungi) in different crust types with protists deserves more attention in future studies.

## Figures and Tables

**Table 1:** Geographical coordinates of biocrust sampling locations.

Country	Locations	Geographic Coordinates	
Germany, Baltic Sea (coastline)	Riedensee	54° 09.179 N	11° 41.431 E
	Heiligendamm	54° 10.816 N	11° 51.346 E
	Warnemünde	54° 10.816 N	12° 04.827 E
	Baabe	54° 21.267 N	13° 43.050 E
	Karlshagen	54° 08.216 N	13° 49.716 E
Iceland	Litla Skardt	64°43′28.884″ N	21°36′49.392″ W
	Fiflholt	64°42′4.968″ N	22°8′24.936″ W
	Krákunes	64°39′21.672″ N	22°20′50.352″ W
	Giljar	64°40′0.804″ N	21°4′8.148″ W
	Borgarfjarðarbrau	64°39′4.82″ N	21°23′40.056″ W

## Data Availability

The raw sequencing data has been deposited in the European Nucleotide Archive under the accession number ERX4911693.

## Supplementary Materials

The following are available online at https://www.mdpi.com/2076-2607/9/2/205/s1, Table S1: Combination of designed barcoded primers targeting Cercozoa and Endomyxa used in this study with the corresponding samples, Table S2: Soil parameters from different biocrust sampling locations. TOC: total organic carbon, N: nitrogen, TP: total phosphorus. Figure S1: Sampling locations in (a) dunes along the coastline of Baltic Sea (Mecklenburg-Western Pomerania, Germany); Riedensee, Heiligendamm, Warnemünde, Baabe and Karlshagen (b) grassland (Iceland); (LSk, Litla Skard; FIF, Fiflholt; KRA, Krákunes; GIL, Giljar; BOR, Borgarfjarðarbraut), Figure S2: Percentages of unique operational taxonomic units (OTUs) per site (grassland and dunes) in the circle and percentages of shared OTUs between two habitats in the intersection, Figure S3: (a) Shannon diversity (H’); (b) Pielou’s evenness (J) of cercozoan and endomyxan OTUs in biocrust samples from different sampling locations in dunes (Germany); Riedensee, Heiligendamm, Warnemünde, Baabe and Karlshagen, Figure S4: (a) Shannon diversity (H’); (b) Pielou’s evenness (J) of cercozoan and endomyxan OTUs in biocrust samples from different sampling locations in grassland (Iceland); (LSk, Litla Skard; FIF, Fiflholt; KRA, Krákunes; GIL, Giljar; BOR, Borgarfjarðarbraut), Figure S5: Correlation of soil parameters (pH, C, N and TP) with the beta diversity community composition of Cercozoa and Endomyxa in biocrusts from: (a) dunes (RS, Riedensee; HD, Heiligendamm; WM, Warnemünde; Bb, Baabe; KH, Karlshagen); (b) grassland (LSk, Litla Skard; FIF, Fiflholt; KRA, Krákunes; GIL, Giljar; BOR, Borgarfjarðarbraut).

## References

[1] Belnap J., Büdel B., Lange O.L., Belnap J., Lange O.L. (2003). Biological soil crusts: Characteristics and distribution. Biological Soil Crusts: Structure, Function and Management.

[2] Darby B.J., Neher D.A., Weber B., Büdel B., Belnap J. (2016). Microfauna within biological soil crusts. Biological Soil Crusts: An Organizing Principle in Drylands.

[3] Campbell S.E. (1979). Soil stabilization by a prokaryotic desert crust: Implications for precamcrian land biota. Orig. Life.

[4] Reynolds R., Belnap J., Reheis M., Lamothe P., Luiszer F. (2001). Aeolian dust in Colorado Plateau soils: Nutrient inputs and recent change in source. Proc. Natl. Acad. Sci. USA.

[5] Elbert W., Weber B., Burrows S., Steinkamp J., Büdel B., Andreae M.O., Pöschl U. (2012). Contribution of cryptogamic covers to the global cycles of carbon and nitrogen. Nat. Geosci..

[6] Belnap J., Prasse R., Harper K.T., Belnap J., Lange O.L. (2003). Influence of biological soil crustson soil environments and vascular plants. Biological Soil Crusts: Structure, Function and Management.

[7] Clarholm M. (1985). Interactions of bacteria, protozoa and plants leading to mineralization of soil nitrogen. Soil Biol. Biochem..

[8] Ekelund F., Rønn R. (1994). Notes on protozoa in agricultural soil with emphasis on heterotrophic flagellates and naked amoebae and their ecology. FEMS Microbiol. Rev..

[9] Bonkowski M. (2004). Protozoa and plant growth: The microbial loop in soil revisited. New Phytol..

[10] Bates S.T., Clemente J.C., Flores G.E., Walters W.A., Parfrey L.W., Knight R., Fierer N. (2013). Global biogeography of highly diverse protistan communities in soil. ISME J..

[11] Geisen S., Tveit A.T., Clark I.M., Richter A., Svenning M.M., Bonkowski M., Urich T. (2015). Metatranscriptomic census of active protists in soils. ISME J..

[12] Urich T., Lanzén A., Qi J., Huson D.H., Schleper C., Schuster S.C. (2008). Simultaneous assessment of soil microbial community structure and function through analysis of the meta-transcriptome. PLoS ONE.

[13] Grossmann L., Jensen M., Heider D., Jost S., Glücksman E., Hartikainen H., Mahamdallie S.S., Gardner M., Hoffmann D., Bass D. (2016). Protistan community analysis: Key findings of a large-scale molecular sampling. ISME J..

[14] Fiore-Donno A.M., Richter-Heitmann T., Degrune F., Dumack K., Regan K., Mahran S., Boeddinghaus R., Rillig M., Friedrich M.W., Kandeler E. (2019). Environmental selection and spatiotemporal structure of a major group of soil protists (rhizaria: Cercozoa) in a temperate grassland. Front. Microbiol..

[15] Dumack K., Fiore-Donno A.M., Bass D., Bonkowski M. (2019). Making sense of environmental sequencing data: Ecologically important functional traits of the protistan groups cercozoa and endomyxa (rhizaria). Mol. Ecol. Resour..

[16] Domonell A., Brabender M., Nitsche F., Bonkowski M., Arndt H. (2013). Community structure of cultivable protists in different grassland and forest soils of Thuringia. Pedobiologia.

[17] Bugge Harder C., Rønn R., Brejnrod A., Bass D., Al-Soud W.A., Ekelund F. (2016). Local diversity of heathland cercozoa explored by in-depth sequencing. ISME J..

[18] Degrune F., Dumack K., Fiore-Donno A.M., Bonkowski M., Sosa-Hernández M.A., Schloter M., Kautz T., Fischer D., Rillig M.C. (2019). Distinct communities of cercozoa at different soil depths in a temperate agricultural field. FEMS Microbiol. Ecol..

[19] Fiore-Donno A.M., Rixen C., Rippin M., Glaser K., Samolov E., Karsten U., Becker B., Bonkowski M. (2017). New barcoded primers for efficient retrieval of cercozoan sequences in high-throughput environmental diversity surveys, with emphasis on worldwide biological soil crusts. Mol. Ecol. Resour..

[20] Khanipour Roshan S., Dumack K., Bonkowski M., Karsten U., Glaser K. (2020). Stramenopiles and Cercozoa dominate the heterotrophic protist community of biological soil crusts irrespective of edaphic factors. Pedobiologia.

[21] Arnalds O. (2005). Icelandic soils. Dev. Quat. Sci..

[22] Arnalds O. (2008). Soils of Iceland. JÖKULL.

[23] Miller T.E., Gornish E.S., Buckley H.L. (2010). Climate and coastal dune vegetation: Disturbance, recovery, and succession. Plant Ecol..

[24] Novo F., Díaz M., Zunzunegui M., Mora R., Gallego-Fernández J., Martínez M.L., Psuty N.P. (2004). Plant functional types in coastal dune habitats. Coastal Dunes: Ecology and Conservation.

[25] McLachlan A., Brown A.C. (2006). The Ecology of Sandy Shores.

[26] Vázquez G., Martínez M.L., Psuty N.P. (2008). The role of algal mats on community succession in dunes and dune slacks. Coastal Dunes: Ecology and Conservation.

[27] Deutsche Wetterdienst, DWD. https://www.dwd.de/.

[28] Iceland Met Office Climatological Data. https://en.vedur.is/climatology/data/.

[29] Pushkareva E., Baumann K., Van T.A., Mikhailyuk T., Baum C., Hrynkiewicz K., Thiem D., Köpcke T., Karsten U., Leinweber P. (2020). Phosphorus turnover and diversity of microbial phototrophs and heterotrophs in Icelandic biocrusts. Geoderma.

[30] Fiore-Donno A.M., Richter-Heitmann T., Bonkowski M. (2020). Contrasting responses of protistan plant parasites and phagotrophs to ecosystems, land management and soil properties. Front. Microbiol..

[31] Schloss P.D., Westcott S.L., Ryabin T., Hall J.R., Hartmann M., Hollister E.B., Lesniewski R.A., Oakley B.B., Parks D.H., Robinson C.J. (2009). Introducing mothur: Open-source, platform-independent, community-supported software for describing and comparing microbial communities. Appl. Environ. Microbiol..

[32] Camacho C., Coulouris G., Avagyan V., Ma N., Papadopoulos J., Bealer K., Madden T.L. (2009). BLAST+: Architecture and applications. BMC Bioinform..

[33] Guillou L., Bachar D., Audic S., Bass D., Berney C., Bittner L., Boutte C., Burgaud G., De Vargas C., Decelle J. (2013). The protist ribosomal reference database (PR2): A catalog of unicellular eukaryote small sub-unit rRNA sequences with curated taxonomy. Nucleic Acids Res..

[34] Edgar R.C., Haas B.J., Clemente J.C., Quince C., Knight R. (2011). UCHIME improves sensitivity and speed of chimera detection. Bioinformatics.

[35] Rognes T., Flouri T., Nichols B., Quince C., Mahé F. (2016). VSEARCH: A versatile open source tool for metagenomics. PeerJ.

[36] Blume H.P., Stahr K., Leinweber P. (2010). Bodenkundliches Praktikum.

[37] Baumann K., Glaser K., Mutz J.E., Karsten U., Maclennan A., Hu Y., Michalik D., Kruse J., Eckhardt K.U., Schall P. (2017). Biological soil crusts of temperate forests: Their role in P cycling. Soil. Biol. Biochem..

[38] Berthold M., Zimmer D., Schumann R. (2015). A simplified method for total phosphorus digestion with potassium persulphate at sub-boiling temperatures in different environmental samples. Rostocker Meeresbiol. Beitr..

[39] R Development Core Team (2019). R: A Language and Environment for Statistical Computing.

[40] Warnes G.R., Bolker B., Bonebakker L., Gentleman R., Liaw W.H.A., Lumley T., Maechler M., Magnusson A., Moeller S., Schwartz M. Gplots: Various R Programming Tools for Plotting Data. https://CRAN.R-project.org/web/packages/gplots.

[41] Oksanen J., Blanchet F., Kindt R., Legendre P., Minchin P., O’Hara R., Simpson G., Solymos P., Stevens M., Wagner H. Vegan: Community Ecology. http://CRAN.R-project.org/package=vegan.

[42] Bray J.R., Curtis J.T. (1957). An ordination of the upland forest communities of southern Wisconsin. Ecol. Monogr..

[43] Bass D., Richards T.A., Matthai L., Marsh V., Cavalier-Smith T. (2007). DNA evidence for global dispersal and probable endemicity of protozoa. BMC. Evol. Biol..

[44] Aguilar M., Lado C. (2012). Ecological niche models reveal the importance of climate variability for the biogeography of protosteloid amoebae. ISME J..

[45] Howe A.T., Bass D., Vickerman K., Chao E.E., Cavalier-Smith T. (2009). Phylogeny, taxonomy, and astounding genetic diversity of glissomonadida ord. nov., the dominant gliding zooflagellates in soil (protozoa: Cercozoa). Protist.

[46] Lentendu G., Wubet T., Chatzinotas A., Wilhelm C., Buscot F., Schlegel M. (2014). Effects of long-term differential fertilization on eukaryotic microbial communities in an arable soil: A multiple barcoding approach. Mol. Ecol..

[47] Griffiths B., Spilles A., Bonkowski M. (2012). C:N:P stoichiometry and nutrient limitation of the soil microbial biomass in a grazed grassland site under experimental P limitation or excess. Ecol. Process..

[48] Griffiths R.I., Thomson B.C., James P., Bell T., Bailey M., Whiteley A.S. (2011). The bacterial biogeography of British soils. Environ. Microbiol..

[49] Trap J., Bonkowski M., Plassard C., Villenave C., Blanchart E. (2016). Ecological importance of soil bacterivores for ecosystem functions. Plant. Soil..

[50] Fierer N., Strickland M.S., Liptzin D., Bradford M.A., Cleveland C.C. (2009). Global patterns in belowground communities. Ecol. Lett..

[51] Dequiedt S., Saby N.P., Lelievre M., Jolivet C., Thioulouse J., Toutain B., Arrouays D., Bispo A., Lemanceau P., Ranjard L. (2011). Biogeographical patterns of soil molecular microbial biomass as influenced by soil characteristics and management. Glob. Ecol. Biogeogr..

[52] Büdel B., Colesie C., Green T.G.A., Grube M., Lázaro Suau R., Loewen-Schneider K., Maier S., Peer T., Pintado A., Raggio J. (2014). Improved appreciation of the functioning and importance of biological soil crusts in Europe: The soil crust international project (SCIN). Biodivers. Conserv..

[53] Dojani S., Kauff F., Weber B., Büdel B. (2014). Genotypic and phenotypic diversity of cyanobacteria in biological soil crusts of the succulent karoo and nama karoo of southern Africa. Microb. Ecol..

[54] Patzelt D.J., Hodač L., Friedl T., Pietrasiak N., Johansen J.R. (2014). Biodiversity of soil cyanobacteria in the hyper-arid Atacama Desert, Chile. J. Phycol..

[55] Schulz K., Mikhailyuk T., Dreßler M., Leinweber P., Karsten U. (2016). Biological soil crusts from coastal dunes at the Baltic Sea: Cyanobacterial and algal biodiversity and related soil properties. Microb. Ecol..

[56] Glaser K., Baumann K., Leinweber P., Mikhailyuk T., Karsten U. (2018). Algal richness in BSCs in forests under different management intensity with some implications for P cycling. Biogeosciences.

[57] Howe A.T., Bass D., Scoble J.M., Lewis R., Vickerman K., Arndt H., Cavalier-Smith T. (2011). Novel cultured protists identify deep-branching environmental DNA clades of cercozoa: New genera tremula, micrometopion, minimassisteria, nudifila, peregrinia. Protist.

[58] Bonkowski M., Villenave C., Griffiths B. (2009). Rhizosphere fauna: The functional and structural diversity of intimate interactions of soil fauna with plant roots. Plant Soil..

[59] Dumack K., Müller M.E.H., Bonkowski M. (2016). Description of lecythium terrestris sp. nov. (chlamydophryidae, cercozoa), a soil dwelling protist feeding on fungi and algae. Protist.

[60] Dumack K., Flues S., Hermanns K., Bonkowski M. (2017). Rhogostomidae (cercozoa) from soils, roots and plant leaves (arabidopsis thaliana): Description of rhogostoma epiphylla sp. nov. and R. cylindrica sp. nov. Eur. J. Protistol..

[61] Dumack K., Mausbach P., Hegmann M., Bonkowski M. (2017). Polyphyly in the thecate amoeba genus lecythium (chlamydophryidae, tectofilosida, cercozoa), redescription of its type species L. hyalinum, description of L. jennyae sp. nov. and the establishment of fisculla gen. nov. and fiscullidae fam. nov. Protist.

[62] Dumack K., Baumann C., Bonkowski M. (2016). A bowl with marbles: Revision of the thecate amoeba genus lecythium (chlamydophryidae, tectofilosida, cercozoa, rhizaria) including a description of four new species and an identification key. Protist.

[63] Dumack K., Öztoprak H., Rüger L., Bonkowski M. (2017). Shedding light on the polyphyletic thecate amoeba genus plagiophrys: Transition of some of its species to rhizaspis (tectofilosida, thecofilosea, cercozoa) and the establishment of sacciforma gen. nov. and rhogostomidae fam. nov. (cryomonadida, thecofilos. Protist.

[64] Dumack K., Pundt J., Bonkowski M. (2018). Food choice experiments indicate selective fungivorous predation in fisculla terrestris (thecofilosea, cercozoa). J. Eukaryot. Microbiol..

[65] Seppey C.V.W., Singer D., Dumack K., Fournier B., Belbahri L., Mitchell E.A.D., Lara E. (2017). Distribution patterns of soil microbial eukaryotes suggests widespread algivory by phagotrophic protists as an alternative pathway for nutrient cycling. Soil Biol. Biochem..

[66] Öztoprak H., Walden S., Heger T., Bonkowski M., Dumack K. (2020). What drives the diversity of the most abundant terrestrial cercozoan family (rhogostomidae, cercozoa, rhizaria)?. Microorganisms.

[67] Jauss R.T., Walden S., Fiore-Donno A.M., Dumack K., Schaffer S., Wolf R., Schlegel M., Bonkowski M. (2020). From forest soil to the canopy: Increased habitat diversity does not increase species richness of cercozoa and oomycota in tree canopies. Front. Microbiol..

[68] Hess S., Sausen N., Melkonian M. (2012). Shedding light on vampires: The phylogeny of vampyrellid amoebae revisited. PLoS ONE.

[69] Hess S. (2017). Hunting for agile prey: Trophic specialisation in leptophryid amoebae (vampyrellida, rhizaria) revealed by two novel predators of planktonic algae. FEMS Microbiol. Ecol..

[70] Old K.M., Darbyshire J.F. (1978). Soil fungi as food for giant amoebae. Soil Biol. Biochem..

[71] Sayre R.M., Wergin W.P. (1989). Morphology and fine structure of the trophozoites of theratromyxa weberi (protozoa: Vampyrellidae) predacious on soil nematodes. Can. J. Microbiol..

[72] Mikhailyuk T., Glaser K., Tsarenko P., Demchenko E., Karsten U. (2019). Composition of biological soil crusts from sand dunes of Baltic Sea coast in the context of an integrative approach to the taxonomy of microalgae and cyanobacteria. Eur. J. Phycol..

[73] Arnalds O. (2015). The Soils of Iceland.

[74] Lara E., Heger T.J., Mitchell E.A.D., Meisterfeld R., Ekelund F. (2007). SSU rRNA reveals a sequential increase in shell complexity among the euglyphid testate amoebae (rhizaria: Euglyphida). Protist.

[75] Flues S., Bass D., Bonkowski M. (2017). Grazing of leaf-associated cercomonads (protists: Rhizaria: Cercozoa) structures bacterial community composition and function. Environ. Microbiol..

