# Supplementary material for: Taxonomic and Functional Diversity of Heterotrophic Protists (Cercozoa and Endomyxa) from Biological Soil Crusts
Source: Microorganisms. 2021 Jan 20;9(2):205. doi: 10.3390/microorganisms9020205 (PMC7908994; doi:10.3390/microorganisms9020205)
Supplement: Supplementary file 1 [file microorganisms-09-00205-s001.pdf]

microorganisms

MDPI

Supplementary Materials:

Table S1. Combination of designed barcoded primers targeting Cercozoa and Endomyxa used in this study with the corresponding samples.

Primer	GTTAAAARGCTCGTAGTYG	AAGARGAYATCCTTGGTG	Sample ID	Sample name based on the location
barcode	AACTTAGC	TACGCTAT	Hd_1	Heiligendamm
barcode	AACTTAGC	GTCAGTAT	Hd_2	Heiligendamm
barcode	AATTGGTC	GCTTCAAT	Hd_3	Heiligendamm
barcode	GTAGATAC	AATCAGGT	Hd_4	Heiligendamm
barcode	GACCATAC	ACGATCAG	Wm_1	Warnemünde
barcode	GACCATAC	CGTTCAAG	Wm_2	Warnemünde
barcode	GACCATAC	TCTCTCAG	Wm_3	Warnemünde
barcode	GTAGATAC	GCTTCAAT	Wm_4	Warnemünde
barcode	GACCATAC	TACTGTAG	RS_1	Riedensee
barcode	TATCTAGC	CCATTATG	RS_2	Riedensee
barcode	TATCTAGC	AATCAGGT	RS_3	Riedensee
barcode	GTTATAGC	TCTCTCAG	RS_4	Riedensee
barcode	TATCTAGC	GCTTCAAT	Kh_1	Karlshagen
barcode	TATCTAGC	TATCAGTC	Kh_2	Karlshagen
barcode	GAATACTC	GTGTCAAC	Kh_3	Karlshagen
barcode	AGTTCATC	GACATATC	Kh_4	Karlshagen
barcode	GAATACTC	ATTCTCGG	Bb_1	Baabe
barcode	GAATACTC	AAGCTACT	Bb_2	Baabe
barcode	TGATACAC	GTAACATG	Bb_3	Baabe
barcode	AGTTCATC	TTAGGAAC	Bb_4	Baabe
barcode	AACTTAGC	ACGATCAG	1_1_I	Fiflholt (FIF)
barcode	AACTTAGC	CGTTCAAG	1_2_I	Fiflholt (FIF)

barcode	TTAGAGTC	AATTGGTC	1_3_I	Fiflholt (FIF)
barcode	TTAGAGTC	ACAATGTG	1_4_I	Fiflholt (FIF)
barcode	TTAGAGTC	CCATTATG	1_5_I	Borgarfjarðarbraut (BOR)
barcode	TTAGAGTC	AATCAGGT	1_6_I	Borgarfjarðarbraut (BOR)
barcode	TTAGAGTC	GCTTCAAT	1_7_I	Borgarfjarðarbraut (BOR)
barcode	TTAGAGTC	TATCAGTC	1_8_I	Borgarfjarðarbraut (BOR)
barcode	TATCTAGC	GTGTCAAC	1_9_I	Krákunes (KRA)
barcode	AATTGGTC	AATTGGTC	1_10_I	Krákunes (KRA)
barcode	TATATGCC	TACTGTAG	1_11_I	Krákunes (KRA)
barcode	TATATGCC	GACATATC	1_12_I	Krákunes (KRA)
barcode	TATATGCC	GTAACATG	1_13_I	Giljar (GIL)
barcode	TATATGCC	TTAGGAAC	1_14_I	Giljar (GIL)
barcode	TATATGCC	TACGCTAT	1_15_I	Giljar (GIL)
barcode	TATATGCC	GTCAGTAT	1_16_I	Giljar (GIL)
barcode	TGATACAC	TTAGGAAC	1_17_I	Litla Skard (LSK)
barcode	TGATACAC	TACGCTAT	1_18_I	Litla Skard (LSK)
barcode	TGATACAC	GTCAGTAT	1_19_I	Litla Skard (LSK)
barcode	TGATACAC	ACGATCAG	1_20_I	Litla Skard (LSK)

Table S2. Soil parameters from different biocrust sampling locations. TOC: total organic carbon, N: nitrogen, TP: total phosphorus.

		Soil parameters					
Country/habitat	Locations	pH	TOC ($mg\ g^{-1}$)	N ($mg\ g^{-1}$)	TP ($mg\ g^{-1}$)	C:N	C:P
Germany, coastal dunes	Riedensee	7.30	4.03	0.41	0.09	9.83	51.55
	Heiligendamm	7.30	2.64	0.27	0.07	9.01	54.25
	Warnemünde	8.06	4.74	0.25	0.08	18.70	70.73
	Baabe	8.40	1.62	0.23	0.12	6.47	20.77
	Karlshagen	8.05	2.23	0.21	0.07	10.93	29.17

Iceland,	Litla Skard	5.53	64.54	5.28	0.89	11.78	67.44
grassland	Fiflholt	5.30	83.20	6.25	0.85	13.15	83.83
	Krákunes	5.73	109.28	6.76	1.27	15.29	85.06
	Giljar	5.78	71.19	6.31	1.23	11.27	54.81
	Borgarfjarðarbraut	5.50	32.55	3.01	1.31	10.17	27.80

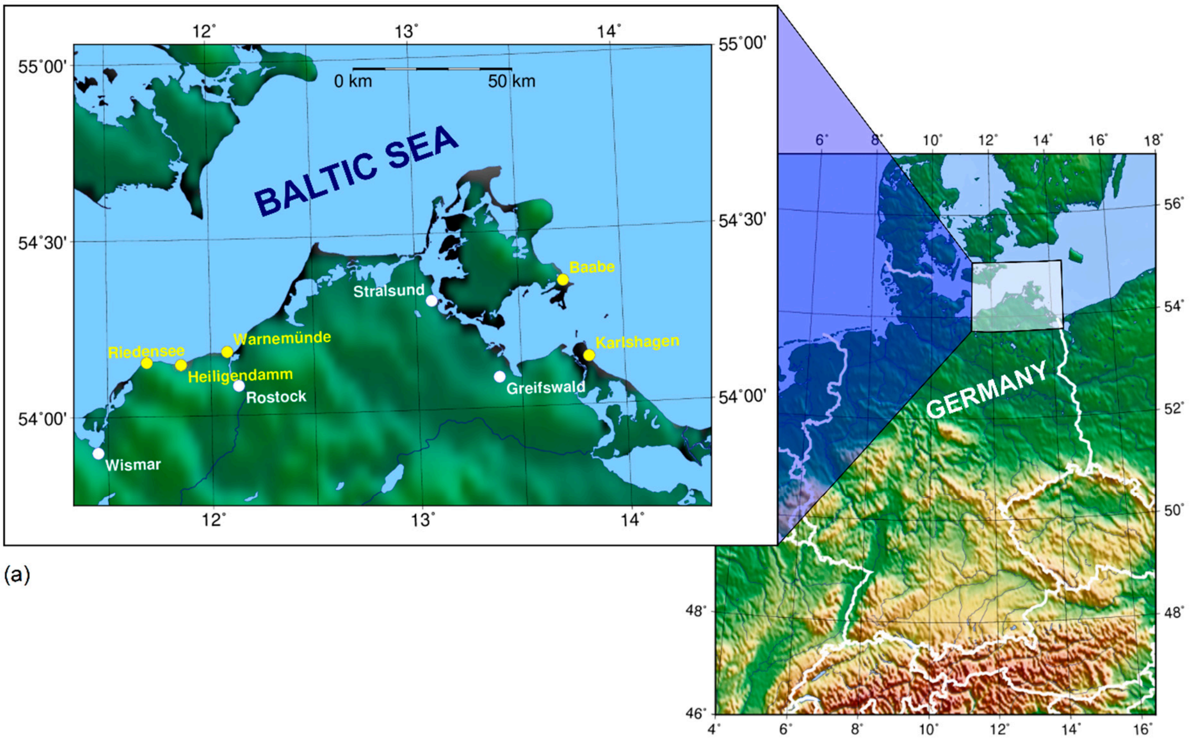

(a)

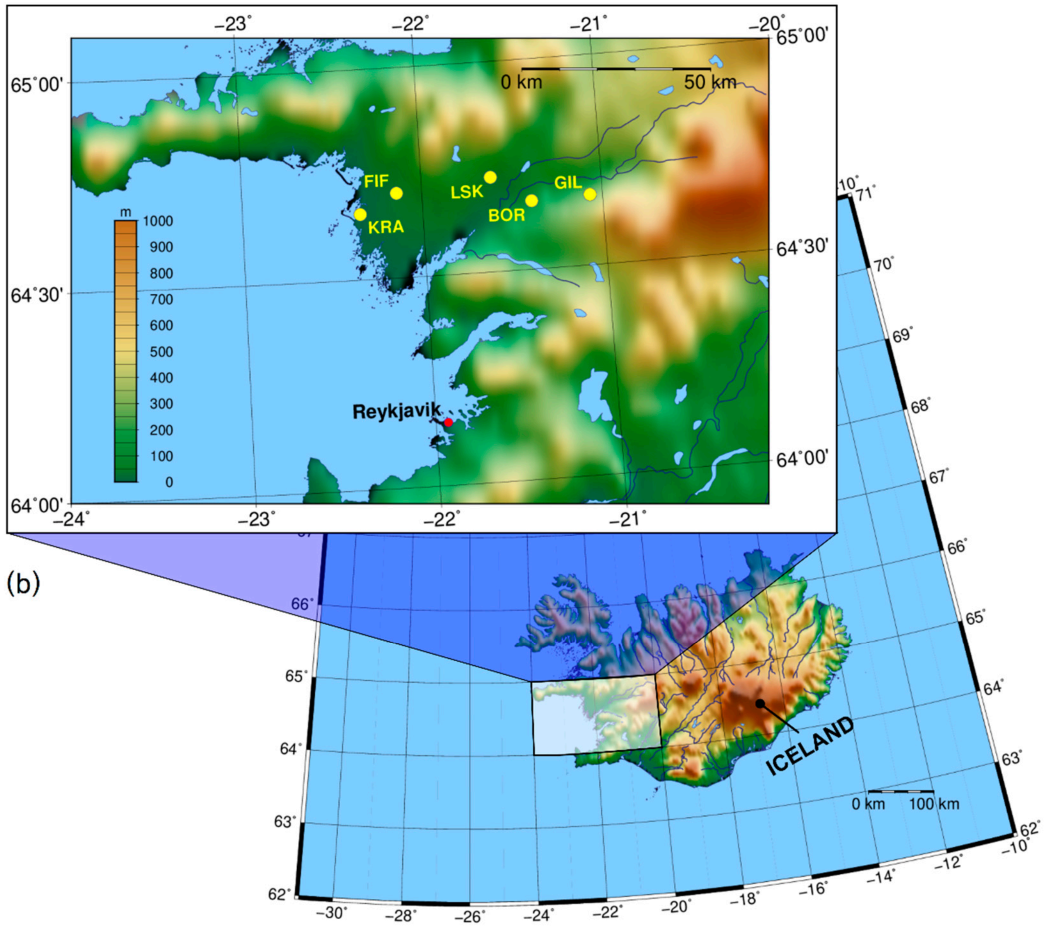

Figure S1. Sampling locations in (a) dunes along the coastline of Baltic Sea (Mecklenburg-Western Pomerania, Germany); Riedensee, Heiligendamm, Warnemünde, Baabe and Karlshagen (b) grassland (Iceland); (LSk, Litla Skard; FIF, Fiflholt; KRA, Krákunes; GIL, Giljar; BOR, Borgarfjarðarbraut).

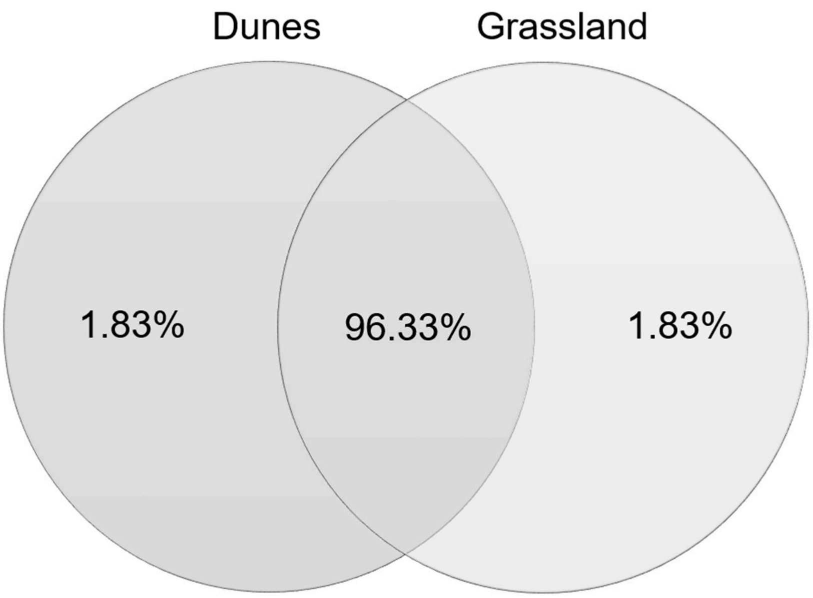

Figure S2. Percentages of unique operational taxonomic units (OTUs) per site (grassland and dunes) in the circle and percentages of shared OTUs between two habitats in the intersection.

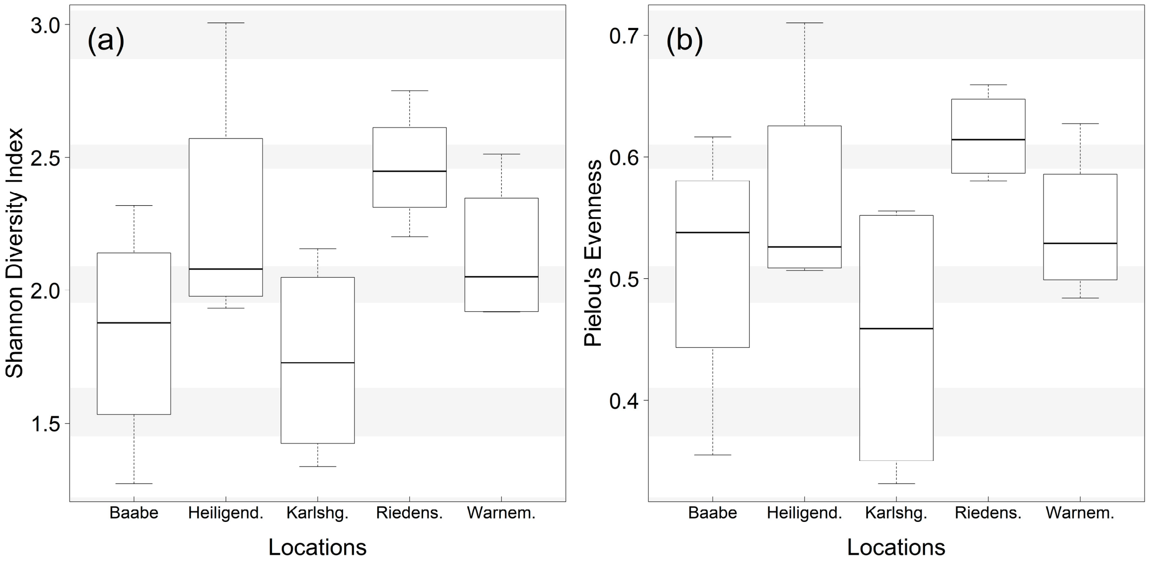

Figure S3. (a) Shannon diversity (H´) and (b) Pielou's evenness (J) of cercozoan and endomyxan OTUs in biocrust samples from different sampling locations in dunes (Germany); Riedensee, Heiligendamm, Warnemünde, Baabe and Karlshagen.

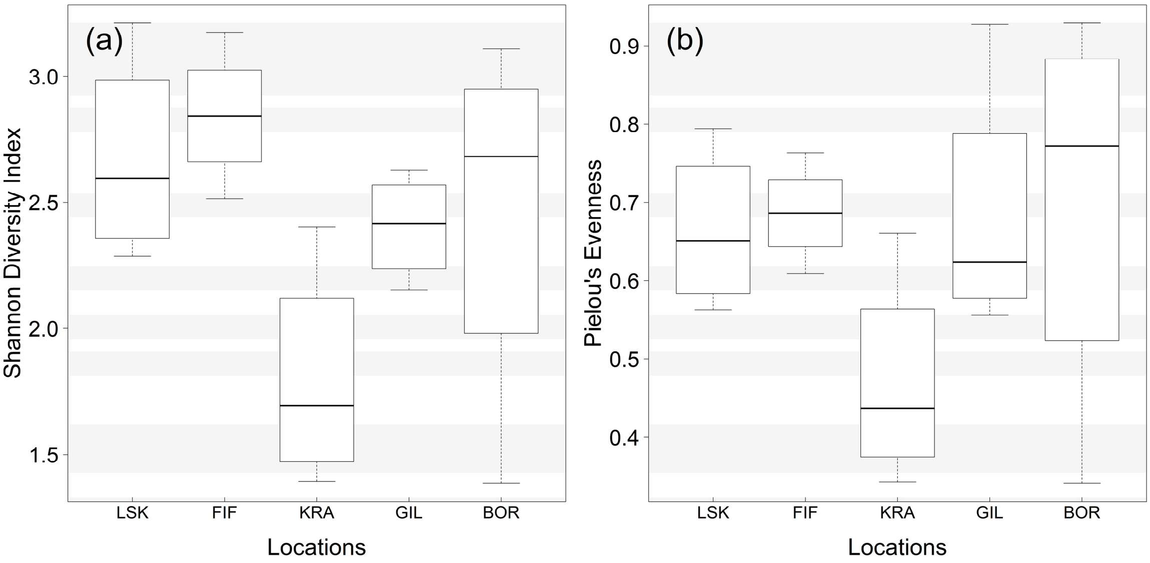

Figure S4. (a) Shannon diversity (H´) and (b) Pielou's evenness (J) of cercozoan and endomyxan OTUs in biocrust samples from different sampling locations in grassland (Iceland); (LSk, Litla Skard; FIF, Fiflholt; KRA, Krákunes; GIL, Giljar; BOR, Borgarfjarðarbraut).

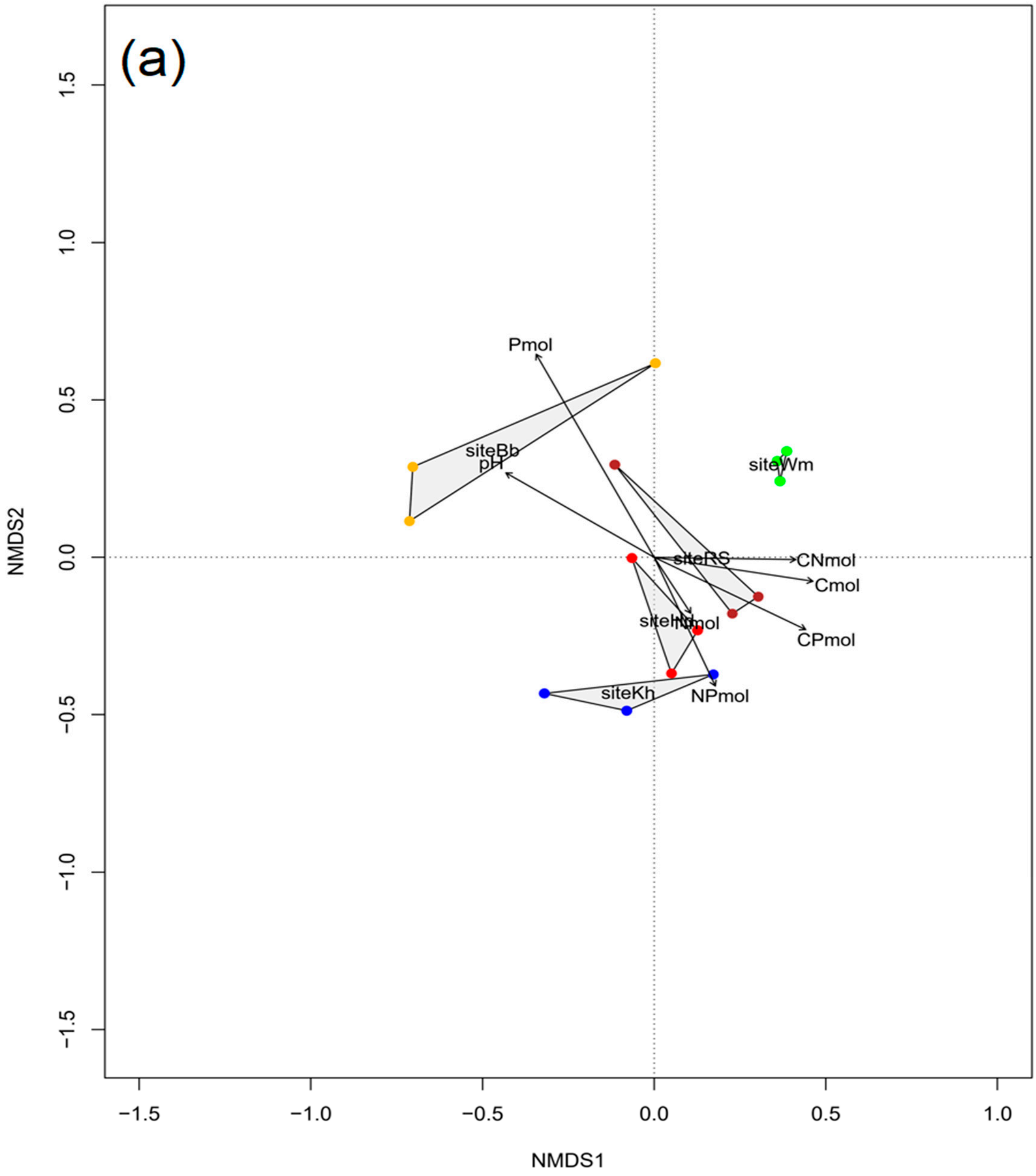
(a)
Pmol
siteBb
pH
siteWm
siteRS
CNmol
Cmol
CPmol
siteHd
Nmol
siteKh
NPmol
NMDS1
NMDS2
1.5
1.0
0.5
0.0
−0.5
−1.0
−1.5
−1.5
−1.0
−0.5
0.0
0.5
1.0

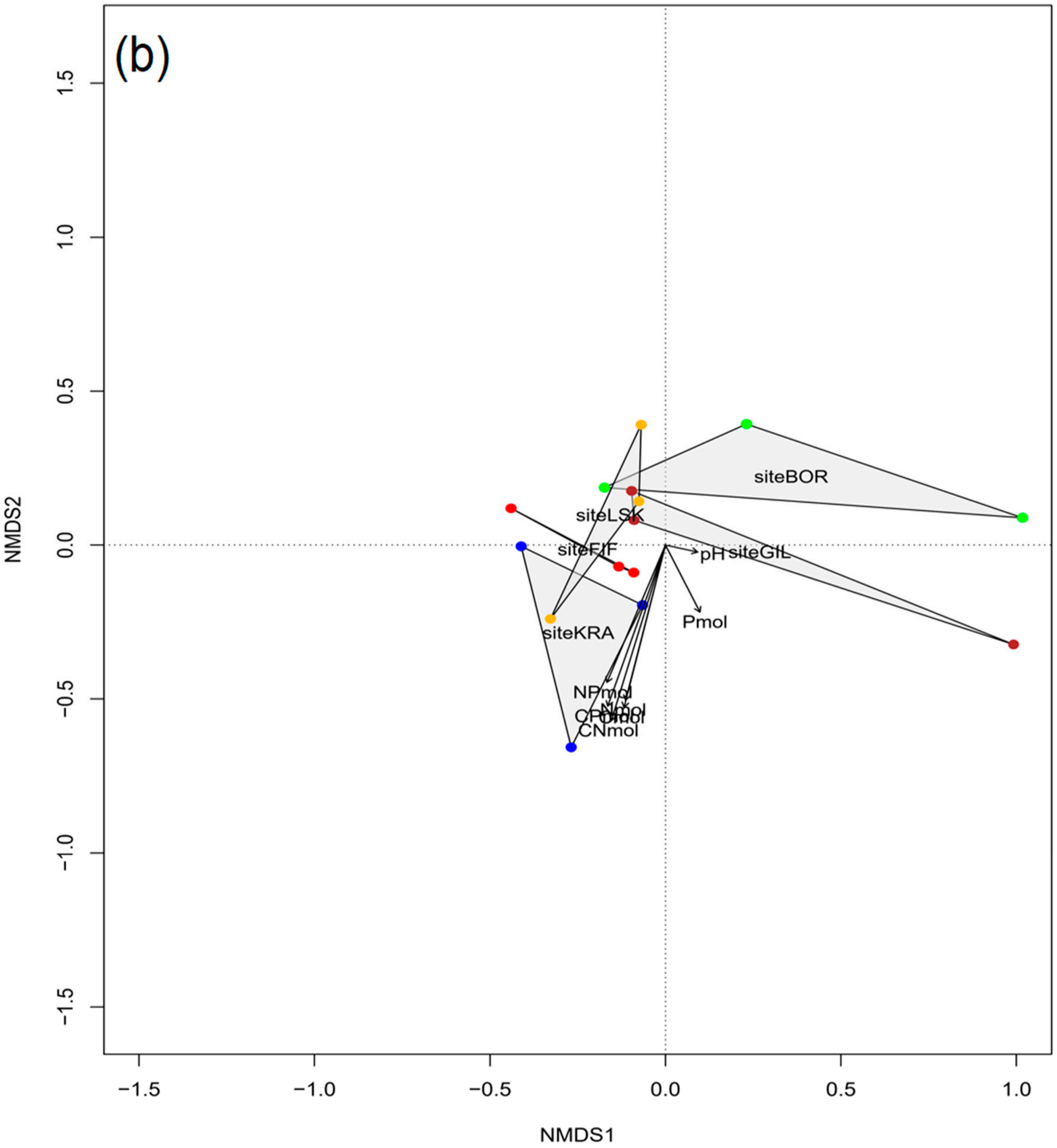

Figure S5. Correlation of soil parameters (pH, C, N and TP) with the beta diversity community composition of Cercozoa and Endomyxa in biocrusts from (a) dunes (RS, Riedensee; HD, Heiligendamm; WM, Warnemünde; Bb, Baabe; KH, Karlshagen) and (b) grassland (LSk, Litla Skard; FIF, Fiflholt; KRA, Krákunes; GIL, Giljar; BOR, Borgarfjarðarbraut).

© 2020 by the authors. Submitted for possible open access publication under the terms and conditions of the Creative Commons Attribution (CC BY) license (http://creativecommons.org/licenses/by/4.0/).
